# BrowseVCF: a web-based application and workflow to quickly prioritize disease-causative variants in VCF files

**DOI:** 10.1093/bib/bbw054

**Published:** 2016-07-02

**Authors:** Silvia Salatino, Varun Ramraj

**Affiliations:** The Wellcome Trust Centre for Human Genetics, Roosevelt Drive, Oxford, UK

**Keywords:** VCF, variant filtering, variant analysis, exome sequencing, whole-genome sequencing, prioritization

## Abstract

Following variant calling and annotation, accurate variant filtering is a crucial step to extract meaningful information from sequencing data and to investigate disease aetiology. However, the variant call format (VCF) used to store this information is not easy to handle for non-bioinformaticians. We present BrowseVCF, a flexible and intuitive software to enable researchers to browse and filter millions of variants in a few seconds. Key features include querying user-defined gene lists, grouping samples for family or tumour/normal studies and exporting results in spreadsheet format. BrowseVCF’s significant advantages over most existing tools include the ability to process data from any DNA sequencing experiment (exome, whole-genome and amplicons) and to correctly parse files annotated with Variant Effect Predictor. BrowseVCF can be used either locally on personal computers or as part of automated pipelines. Its user interface has been carefully designed to minimize tunable parameters. BrowseVCF is freely available from https://github.com/BSGOxford/BrowseVCF/releases/latest.

## Introduction

Recent developments of Next-Generation Sequencing (NGS) technologies have led to a dramatic reduction in sequencing costs that, in turn, made DNA sequencing analyses accessible to small- and medium-sized laboratories. Consequently, increasing amounts of sequencing data need to be analysed quickly and require specific bioinformatics expertise. While large institutes might be able to rely on support from dedicated bioinformatics core units, wet-lab researchers from smaller centres may struggle to find easy-to-use tools for variant analysis. The variant call format (VCF), originally developed for the 1000 Genomes Project, has become the standard for storing DNA variants together with rich annotations [1], and has led to the development of a variety of command-line analysis tools (for instance, VCFtools [1] or GEMINI [2]). Recent efforts have been made towards developing more user-friendly graphical software to increase accessibility to non-bioinformaticians; examples include the commercial suites Ingenuity (http://www.ingenuity.com/), Alamut (http://www.interactive-biosoftware.com/alamut-visual/), GoldenHelix SNP (http://goldenhelix.com/SNP_Variation/index.html) and VariantStudio (http://www.illumina.com/informatics/research/biological-data-interpretation/variantstudio.html), as well as the open-source packages SNVerGUI [3], database.bio [4] and gNOME [5]. However, a disadvantage common to all these tools is that they strongly depend on a well-defined set of annotations, which limits the user to a restricted number of pre-defined features. Instead, researchers might be interested in keeping their personal or in-house annotations for downstream analysis, rather than discarding them. To our knowledge, only one recently published software (VCF-Miner) addresses this problem by skipping the annotation step and focusing on the filtering part [6]. However, its installation process requires administrative privileges and knowledge of virtual machines or Docker containers, it does not natively handle Variant Effect Predictor (VEP, http://www.ensembl.org/info/docs/tools/vep/index.html) annotations and it does not allow the user to query variants that are annotated with a given set of genes of interest. Finally, certain variant filtering tools, like BiERapp [7] and exomeSuite [8], are optimised purely for exome data, rather than much larger whole-genome data sets.

BrowseVCF overcomes these and other limitations, providing a faster, portable and simpler interactive analysis tool to non-bioinformatician researchers. It has been optimized to reduce VCF loading and indexing time as much as possible by allowing the user to select only the fields of interest. The filter-history export feature was carefully designed to produce an easy-to-read report for posterity and reproducibility. It runs natively on multiple operating systems without the need for administrative privileges or knowledge of sophisticated deployment models. Moreover, our software has a new feature that allows users to perform keyword-based searches to identify, for instance, specific consequence terms or associated disorder states.

By applying filters sequentially to the input set of variants, BrowseVCF empowers researchers to narrow down the amount of putative variants to a small number of candidate disease-causing alleles.

## Materials and methods

### VCF file loading and pre-processing

The VCF file format is very well-defined [1]. It consists of a header section, containing an arbitrary number of meta-information lines that start with the symbol `#', and of a data section, containing one line per variant, split into eight mandatory columns: chromosome (CHROM), 1-based starting position of the variant (POS), unique identifier, if existing (ID), reference allele in the genome (REF), alternative allele(s) of the variant (ALT), Phred-scaled quality score (QUAL), flag for passed/failed control checks (FILTER) and variant-specific annotations (INFO), which can be an unrestricted number of either flags (present/absent) or key-value pairs. VCF files containing one or more samples also include a ninth column (FORMAT), used to define the information enclosed in each subsequent column, and a genotype column for each sample, regarding the allele combination, the genotype read depth and other metrics.

**Figure 1.**
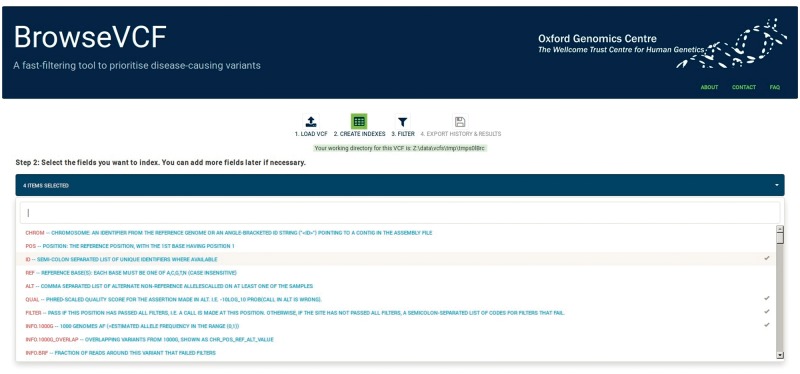
Screenshot of BrowseVCF step 2 (index creation). The drop-down menu lets the user browse through the annotation fields present in the input VCF file and select one or more fields to be used in filtering. A text box allows keyword searching across fields.

The loading step within BrowseVCF is straightforward. The user selects a VCF file from his personal computer through a pop-up dialog box, which is then pre-processed. This step is able to correctly parse a variety of annotations, including those generated by VEP. BrowseVCF can accept both uncompressed and compressed (*.gz) VCF file types. The software identifies every annotation field present in the input VCF file and presents this list to the user, who can then select the fields of interest that will be used to filter the variants (Figure 1). Additional fields can be selected at any time, if needed.

The ‘Wormtable’ Python module [9] was chosen over other data storage systems for its efficient use of disk space and for the speed of its queries. However, as one Wormtable is created per chosen field, selecting fewer fields will reduce the time it takes for BrowseVCF to process the file. The pre-processing step is also required to convert missing values (`.') to `nan' and to convert “CSQ” annotations (in case the file was annotated with VEP) into a Wormtable-compatible format.

### Variant prioritization by sequential filtering

**Figure 2.**
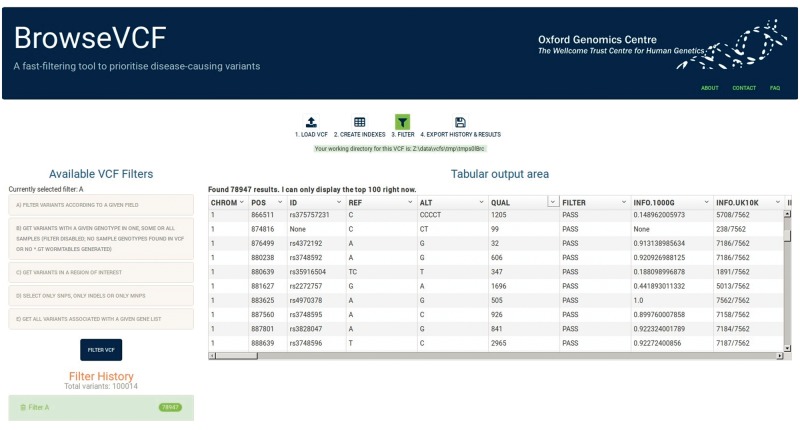
Screenshot of BrowseVCF step 3 (filtering). The left panel lists the five available filters, and expands to allow the user to define various filter options and cut-offs. The ‘Filter History’ panel keeps track of all the sequential filters applied to the initial data and shows the output number of variants. The right panel is the output area, which displays the top 100 variants resulting from each consecutive filter. Fields (shown as columns) can be sorted or hidden if desired.

Once the file is pre-processed, the user can start to filter variants according to five different query types (Figure 2). The first filter lets the user query the data based on a given field of interest. Based on the type of annotation, different operators can be chosen for a specific cut-off/keyword (‘greater_than’, ‘less_than’, ‘equal_to’ or ‘contains_keyword’) and the user can choose whether to include or exclude variants with missing values. Moreover, the ‘contains_keyword’ option allows searching for multiple keywords within the same field, which proves useful when the objective is to keep variants labelled with different consequence or disease tags, for example. The second filter extracts variants with a given genotype in one, some or all samples; moreover, it is able to distinguish between ‘homozygous reference’, ‘homozygous alternate’ and ‘heterozygous’. This feature is particularly useful in the study of inherited and *de novo* syndromes within a family or close relatives. BrowseVCF is able to process both diploid and non-diploid genomes alike. The third filter can retrieve all variants located within a given region of interest; in this case, the user is prompted to specify chromosome, start position and end position. This option is particularly useful to integrate other sources of information, e.g. regions identified by linkage analysis or targeted for capture. The fourth filter is able to extract Single-Nucleotide Polymorphisms (SNPs), Insertions-Deletions (InDels), or Multi-Nucleotide Polymorphisms (MNPs). Finally, the fifth query allows the user to specify a list of genes and retrieves all variants that are either associated (positive query) or not (negative query) to any of those entries within a specific annotation field. Filters are applied sequentially, so that a new filter is applied only to the variants that have successfully passed the previous filter.

### Exporting results from the graphical interface

BrowseVCF comes with a user-friendly graphical interface (GUI) that allows researchers to load, filter, track and export variants and annotations. Figure 2 displays the filtering interface. The right panel of the GUI displays variants in a tabular form, whereas the left panel keeps track of the filters applied and of the number of variants resulting from each query. The user can download the query history for record-keeping at any point. The output of each query is automatically stored to disk and can be deleted at any time from the ‘Filter History’ section in the left panel. The right panel shows a preview of the top 100 variants and all variants can be exported at any time as a tab-separated text file, compatible with spreadsheet programs like Microsoft Excel.

### Technical details

BrowseVCF is a free software under the GNU General Public License version 3 (http://www.gnu.org/licenses/gpl-3.0.en.html). It consists of a back-end service of Python scripts and a web server that runs locally, and of a front end built in JavaScript, CSS and HTML5, leveraging popular cross-browser libraries such as AngularJS and Bootstrap. It is bundled with a sample VCF file and a PDF version of the tutorial. To perform fast and memory-efficient queries, all filtering operations are executed using Wormtable, a recently developed Python module optimized to store genomic data in a compact binary tabular format through Berkeley DB, and retrieve data quickly by indexing desired fields [9]. It is this use of Wormtable for data storage that makes BrowseVCF powerful, as future filters and enhancements can be implemented relatively easily by amending the indexes that are created in the Wormtable. The BerkeleyDB library is approximately 300 kB, but can operate on up to 256 TB of data in theory, satisfying our scalability criteria and making Wormtable very easy to package.

The back-end scripts pre-process input VCFs and provide an interface to communicate with Wormtable in an intuitive manner, both on the command-line and through the web interface. To enable cross-platform usage, especially on Windows, Wormtable was altered and optimized. Every operation is carried out locally on the user's computer, using the default browser; BrowseVCF is supported by all modern browsers, including Firefox and Chrome. The Windows version of BrowseVCF is shipped along with a lightweight distribution of WinPython, a bundled environment pre-configured to run directly on Windows. This enables the Windows user to simply double-click on an executable file without needing administrative permissions to install Python and necessary custom modules, which greatly improves the accessibility and ease-of-use of our software. Similarly, Mac-specific and GNU/Linux versions are also provided as bundled distributions. However, as the tool can be configured to run with any web server back-end, it can also be installed site-wide within an organization, thereby enabling shared use. Detailed instructions to compile BrowseVCF from source are provided on GitHub for this purpose.

### Command-line version of BrowseVCF

BrowseVCF can also be used from command-line as a set of one-off queries, or as part of automated analysis pipelines. Each Python script is independent from the others and the list of required arguments can be displayed by typing the script name followed by ‘--help’. Some of the scripts are capable of parallel execution by specifying the number of cores at invocation, allowing BrowseVCF to operate within a high-performance computing (HPC) environment.

### Data access

BrowseVCF is freely available for download from https://github.com/BSGOxford/BrowseVCF/releases/latest

## Results

### Benchmarks

We tested the performance of BrowseVCF on two different file types: an exome trio (proband, mother, father) and the whole-genome v2.18 of sample NA12878 generated by the ‘Genome in a Bottle Consortium’ (https://sites.stanford.edu/abms/giab). As shown in Table 1, the amount of time required to pre-process the VCF file is <2 min for standard exome data and the first query takes usually <30 s. Because each subsequent query uses the filtered output of the previous one, execution time gets shorter. For whole-genome data, pre-processing and index creation time is substantially longer than for an exome VCF (as expected). Timings were collected using a single core and differed between the Windows, GNU/Linux and Mac machines. The table also shows timings taken for a similar tool, VCF-Miner, on the same hardware. We encountered a server error repeatedly when testing VCF-Miner’s Indexing and Filtering steps on the Mac using both Docker and virtual machine deployment options, and as such, were unable to collect this data.

**Table 1:** Performance of BrowseVCF and VCF-Miner on exome and whole-genome data. Pre-processing times vary between operating systems due to implementation differences intrinsic to Python

VCF file	Size (MB)	Variants	Samples	Step 1: Pre-processing^a^			Step 2: Indexing^b^			Step 3: Filtering^c^		
				GNU/ Linux	Windows	Mac OS	GNU/ Linux	Windows	Mac OS	GNU/ Linux	Windows	Mac OS
BrowseVCF												
Exome_trio.vcf.gz	22	100 014	3	0m 18s	0m 22s	0m 23s	0m 35s^d^	1m 2s^d^	1m 11s^d^	0m 14s	0m 28s	0m 25s
GIAB_v2.18.vcf.gz	313	2 915 731	1	5m 8s	6m 29s	6m 45s	9m 11s^d^	37m 53s^d^	39m 16s^d^	3m 38s	13m 9s	11m 48s
1000G_chr22_ 10kVariants.vcf.gz	39	10 000	1092	0m 48s	1m 8s	1m 6s	1m 18s^d^	1m 46s^d^	1m 45s^d^	1m 5s	2m 17s	1m 1s
1000G_chr22_ 20kVariants.vcf.gz	76	20 000	1092	1m 31s	2m 3s	2m 11s	2m 33s^d^	3m16s^d^	3m 34s^d^	1m 56s	2m 27s	2m 3s
VCF-Miner												
Exome_trio.vcf.gz	22	100 014	3	0m 58s	0m 31s	0m 53s	1m 11s	1m 10s	1m 11s	0m 2s	0m 2s	0m 3s
GIAB_v2.18.vcf.gz	313	2 915 731	1	19m 26s	14m 56s	22m 1s	52m 38s	193m 11s	###	0m13s	0m 9s	###
1000G_chr22_ 10kVariants.vcf.gz	39	10 000	1092	0m 40s	0m 37s	0m 40s	0m 10s	0m 8s	0m 15s	0m 2s	0m 2s	0m 3s
1000G_chr22_ 20kVariants.vcf.gz	76	20 000	1092	1m 13s	1m 25s	1m 9s	0m 19s	0m 15s	1m 20s	0m 2s	0m 2s	0m 4s

*Note:* The ‘Windows’ machine had 8 GB RAM and bundled, non-optimized WinPython; the ‘GNU/Linux’ machine had 8 GB RAM and optimized system Python; the ‘Mac’ machine was a MacBook Pro laptop with 16 GB RAM and bundled Python. Other modules and libraries are at identical versions between the two systems. All operations were performed using only 1 CPU.

^a^Step required to convert the input VCF file to a format accepted by Wormtable.

^b^Wormtables generated for the following fields: CHROM + POS, ID, REF + ALT, QUAL, FILTER.

^c^Query executed on the FILTER field, keeping only PASS variants.

^d^These timings can be significantly improved by using multiple cores.

### Data not available (see text).

The bundled WinPython with Windows is not as well optimized as a natively installed Python, and so, Windows performance can be increased by using a different installed Python (this also does not require administrator privileges). Specifying a higher number of CPUs to use in step 2 speeds up the process of creating the Wormtables for the fields of interest.

### Application examples

At the Wellcome Trust Centre for Human Genetics (WTCHG), the variant calling step is followed by an annotation step. This is automated in most cases, but requires custom scripting if the researcher asks for a particular set of annotations to be used. Therefore, as not all VCF files produced by the Oxford Genomics Centre facility contain exactly the same information, it is necessary to have an application able to handle as many VCF annotations as possible. For example, a common analysis in the WTCHG is the joint variant calling on a trio of mother, father and child. In this case, the crucial filter is based on the genotype of the different individuals. Assuming, for instance, that the disease might be caused by a *de novo* germ line mutation in the child, it is possible with BrowseVCF to return all variants in which mother and father are homozygous reference, but the child is heterozygous. Another common example of analysis consists in identifying somatic variants from a matched tumour/normal sample pair from one patient. In this case, as joint variant calling would not report calls that are homozygous reference in both samples, it would be sufficient to set the filter for homozygous reference in the normal sample, to obtain all variants that are either heterozygous or homozygous variant in the tumour sample. For matched tumour/normal sample pairs, it is also useful to discard as putative disease-causing mutations any variant that is frequently observed in large cohort studies. For example, if the VCF file contains allele frequencies (AF) from the 1000 Genomes Project, ExAC or UK10K, BrowseVCF can be used to keep only those variants with <0.01 AF or not found in the database of interest. BrowseVCF is also capable of handling very long character strings of annotation, like the one returned by the VEP. While VEP information could putatively come from several sources, including Ensembl IDs, PolyPhen, amino-acid position or biotype, BrowseVCF does not make any assumptions about the source data. A useful filter to narrow down the number of candidate variants can be applied, for example, to the CSQ_Consequence field; because BrowseVCF enables multi-keyword searches, it is possible to query using a comma-separated list for ‘missense,frameshift,stop_lost,stop_gained’ (only to mention a few high-impact effects from VEP). Finally, if a list of candidate genes of interest or a set of regions of interest is available, BrowseVCF allows querying of a specific field containing gene symbols (in the first case), or selecting only variants located within user-defined intervals. These last examples highlight the importance of multiple query formats and the usefulness of our software to make this possible. As a result, the WTCHG is now able to effectively use BrowseVCF to analyse VCF data, regardless of how and where they were annotated.

## Discussion

The crucial step in variant analysis studies is an accurate prioritization of the identified mutations, to pinpoint a small number of putative candidates that might be causal for a given disease or phenotype. BrowseVCF is a fast and intuitive interactive software that enables clinicians and researchers to browse and query their data without the need of any bioinformatics expertise. The major advantages of BrowseVCF with respect to similar tools are its clean interface and fast implementation, both particularly useful when analysing millions of variants from whole-genome sequencing data. Additionally, BrowseVCF is straightforward to set up on any machine, as it does not require administrator privileges on a personal machine to install and run. However, BrowseVCF shows a propensity for shorter execution times on GNU/Linux machines over Windows and Mac machines of similar specification owing to operating-system-specific differences in the included WinPython.

A limitation of existing tools is the need to load the entire VCF file, even though no more than a dozen annotations may typically be used for the analysis. In contrast, BrowseVCF allows the user to choose which annotations to process before running the filters and, if needed, to go back and load additional fields for further filtering steps. Considering that the number of samples and annotations in VCF files are the main drivers of the load time, being able to restrict the analysis only to the subset of truly relevant fields saves both computational time and disk space.

In addition, our software can be used both interactively and as part of computational pipelines. Furthermore, the ability to perform all the analysis locally is a considerable advantage when dealing with clinically sensitive files. Although web-based interfaces are easier to operate without the need for command-line scripts, they can pose a security risk when dealing with sensitive patient data [10]. We designed BrowseVCF with flexibility in mind: it can run on a personal laptop without administrative privileges, as part of a HPC environment, or (in cases where patient data are available to an organization behind its firewall) deployed centrally as a web service. In cases where data sensitivity is less of an issue, BrowseVCF is deployable as a public online web service. As BrowseVCF's design focuses almost entirely on solving the problem of portability and fast deployment for non-technical users, Wormtable (and by extension, BerkeleyDB) presented an attractive storage and indexing engine for this purpose, as it is small, easy to distribute under a permissive license and can run independently on any machine without the need to provision servers or virtual machines. Furthermore, other NoSQL database engines such as MongoDB or CouchDB often experience performance issues on 32-bit machines, or machines with less RAM or disk space (owing to intricacies of how the database is stored, rate-of-growth of document store formats and other factors outside the scope of this article). BerkeleyDB does not natively suffer from these limitations. In conclusion, BrowseVCF can significantly improve efficiency in navigating large data sets to find candidate disease variants, thereby streamlining the clinical research process.

BrowseVCF is under active development and maintenance. At the time of writing, release version 2.6 is the latest and includes all the features discussed herein. Development and testing continue for implementation of new features, such as saving and resuming sessions, and global configuration options. However, the current version shows stability and robustness on multiple platforms and types of VCF annotations and is ready for deployment in a production environment.

Key Points

- Browse and filter millions of variants from exome and whole-genome sequencing data in a few seconds.
- Group samples for family or tumour/normal studies to investigate disease aetiology.
- Handle annotations generated by any tool, including VEP, and user-defined gene lists.
- Save query-history and export results in spreadsheet format.
- Straightforward installation without need of administrator privileges.

## Notes

**Silvia Salatino**, PhD, is a member of the Oxford Genomics Centre at the Wellcome Trust Centre for Human Genetics. Her research interests are in analysing large-scale genomics data to understand the cause of complex diseases.

## Notes

**Varun Ramraj**, D.Phil, was a member of the Oxford Genomics Centre at the Wellcome Trust Centre for Human Genetics at the time of writing. He is now a Bioinformatics Support Scientist at Illumina Cambridge Ltd.
